# Metabolic profile distinguishes laminitis-susceptible and -resistant ponies before and after feeding a high sugar diet

**DOI:** 10.1186/s12917-021-02763-7

**Published:** 2021-01-28

**Authors:** Julien Delarocque, Dania B. Reiche, Alexandra D. Meier, Tobias Warnken, Karsten Feige, Martin N. Sillence

**Affiliations:** 1 Clinic for Horses, University of Veterinary Medicine Hannover, Foundation, 30559 Hannover, Germany; 2 Boehringer Ingelheim Vetmedica GmbH, 55216 Ingelheim am Rhein, Germany; 3 Biology and Environmental Science School, Queensland University of Technology, Brisbane, Queensland 4000 Australia

**Keywords:** Laminitis, Equine metabolic syndrome, Pituitary pars intermedia dysfunction, Metabolome, Biomarker, Insulin dysregulation

## Abstract

**Background:**

Insulin dysregulation (ID) is a key risk factor for equine endocrinopathic laminitis, but in many cases ID can only be assessed accurately using dynamic tests. The identification of other biomarkers could provide an alternative or adjunct diagnostic method, to allow early intervention before laminitis develops. The present study characterised the metabolome of ponies with varying degrees of ID using basal and postprandial plasma samples obtained during a previous study, which examined the predictive power of blood insulin levels for the development of laminitis, in ponies fed a high-sugar diet. Samples from 10 pre-laminitic (PL – subsequently developed laminitis) and 10 non-laminitic (NL – did not develop laminitis) ponies were used in a targeted metabolomic assay. Differential concentration and pathway analysis were performed using linear models and global tests.

**Results:**

Significant changes in the concentration of six glycerophospholipids (adj. *P* ≤ 0.024) and a global enrichment of the glucose-alanine cycle (adj. *P* = 0.048) were found to characterise the response of PL ponies to the high-sugar diet. In contrast, the metabolites showed no significant association with the presence or absence of pituitary pars intermedia dysfunction in all ponies.

**Conclusions:**

The present results suggest that ID and laminitis risk are associated with alterations in the glycerophospholipid and glucose metabolism, which may help understand and explain some molecular processes causing or resulting from these conditions. The prognostic value of the identified biomarkers for laminitis remains to be investigated in further metabolomic trials in horses and ponies.

**Supplementary Information:**

The online version contains supplementary material available at 10.1186/s12917-021-02763-7.

## Background

Insulin dysregulation (ID) is an endocrine disorder of horses and ponies, characterized by basal and/or postprandial hyperinsulinemia [1]. Prolonged hyperinsulinemia is associated with a high incidence of endocrinopathic laminitis (also known as insulin-associated laminitis), a painful and debilitating hoof condition, which in severe cases can necessitate euthanasia [2, 3]. While it is known that animals affected by ID may also suffer from equine metabolic syndrome (EMS) and/or pituitary *pars intermedia* dysfunction (PPID), the pathogenesis and detailed pathophysiology of ID are yet to be elucidated.

A relatively new approach to understanding complex disease processes is metabolomics [4]. This involves the comprehensive analysis of disparate small molecules involved in cellular processes, and has already been used, for example, to identify metabolic pathways involved in disorders such as human metabolic syndrome or risk factors for the progression towards clinical diseases such as type 2 diabetes [5]. In recent years, metabolomic studies have increasingly been conducted in livestock as well [6]. The metabolic profile during an oral glucose test (OGT) has also been described in horses and ponies [7, 8].

Meanwhile, several models of insulin-associated laminitis have been developed [2, 3, 9, 10]. Among these, Meier et al. [10] described a method to induce laminitis in insulin-dysregulated ponies using a ‘challenge diet’ containing a high level of sugar and other non-structural carbohydrates (NSC). This method offers the advantage of exacerbating a pre-existing metabolic condition, using a natural dietary stimulus that resembles conditions that may be encountered in the field. The model corroborated the positive correlation between serum insulin concentrations and laminitis risk [10], and so several samples from that study were selected for further investigation using the metabolomics approach.

The present study used both basal plasma samples collected after an overnight fast a few days before the dietary challenge period, and postprandial samples collected 90 min after feeding the challenge diet, from ponies that subsequently did and did not develop laminitis. The primary aim of this retrospective study was to identify potential metabolic biomarkers, in addition to insulin, that may be associated with the onset or recurrence of laminitis. A second aim was to examine the effects of feeding in both groups, to determine if the power to predict laminitis was greater in samples from fed or fasted ponies, and/or to determine if feeding is a potential confounding factor. Finally, by identifying specific metabolites that differ in concentration according to laminitis risk, or which respond differently to feeding in both groups, we aimed to increase our understanding of the pathophysiology of ID.

## Results

### Data pre-processing

The 188 metabolites measured using the Biocrates AbsoluteIDQ p180 Kit belong to six substance classes. By summarising these classes and adding the kynurenine to tryptophan ratio (Kyn/Trp), 194 features were obtained. After pre-processing, 132 features were still present. Table 1 summarises the metabolites initially present and included in the analysis for each substance class. The number of samples varied between 10 and 20, depending on the hypothesis of interest. The characteristics of the sample population are described in an additional file (see Additional file 1). Fifteen missing values for threonine (Thr) had to be imputed using the *k*-nearest neighbours method.

**Table 1:** Summary of the metabolites present in the plasma of insulin-dysregulated ponies, grouped by chemical classes, before and after normalisation. Metabolites that were below the limit of detection in > 50% of samples, or which had a coefficient of variation within QC-samples > 20%, are excluded. Summary columns were added (i.e. sum of each class, except for sugars, and kynurenine:tryptophan ratio), resulting in 132 features included in the data analysis

Class	Before normalisation	After normalisation
Acylcarnitines	40	4
Amino acids	21	21
Biogenic amines	21	12
Glycerophospholipids	90	73
Sphingolipids	15	15
Sugars	1	1
Summary values	6	6
Total	194	132

From 188 metabolites, 181 could be associated with a metabolite identifier from the human metabolome database (HMDB) [11]. Of the 126 metabolites (without the summary values) included in the data analysis, 125 were associated with an HMDB identifier. Especially among lipids, one feature can correspond to several isomers because the assay relies on flow-injection analysis for some of the metabolite classes. In such cases the first best match from the HMDB database was used. Sixty-four HMDB identities could then be associated with 1006 unique metabolic pathways from the small molecule pathway database [12]. Twenty-one pathways, including at least three metabolites from the assay, were available for metabolite set enrichment analysis (MSEA).

### Comparisons between pre-laminitic and non-laminitic ponies

#### Basal samples

**Fig. 1:**
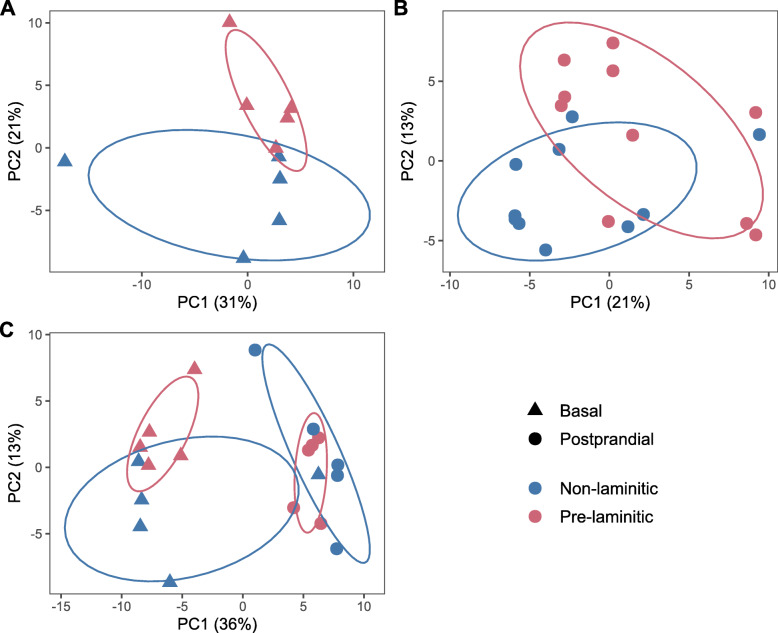
Principal component analysis (PCA) of 132 metabolite concentrations in the plasma of ponies after an overnight fast (basal), or feeding a high-NSC diet (postprandial) from a cohort that contained a group of animals that subsequently developed laminitis (Pre-laminitic, red), and a group that did not (Non-laminitic, blue). To represent high dimensional data, PCA creates a linear combination of the dataset features such as to capture the maximum variance in the data. The amount of total variation explained by the two first principal components is given along the x and y axis, respectively. Each point represents one plasma sample from one pony either in the basal (triangle) or postprandial state (circle). The ellipses represent the 68% confidence interval of each group. The basal laminitic and non-laminitic samples (**a**) fell into two clusters which were best separated by features associated with PC2. In contrast, there was a greater overlap between the groups for the postprandial samples (**b**). When the factors of group and feeding were combined (**c**), some separation between groups was still evident for the basal samples, whereas the groups clustered together in the postprandial samples. A good separation between clusters indicates that it is possible to distinguish both groups by a linear combination of features

Although principal component analysis (PCA) revealed a good separation between PL (*n* = 5) and NL (*n* = 5) ponies for basal samples (Fig. 1a), no significant differences were detected in individual metabolite concentrations (Additional file 2), indicating insufficient discriminatory power of single metabolites at group sizes of *n* = 5. Likewise, no significantly enriched pathways were identified.

#### Postprandial samples

**Fig. 2:**
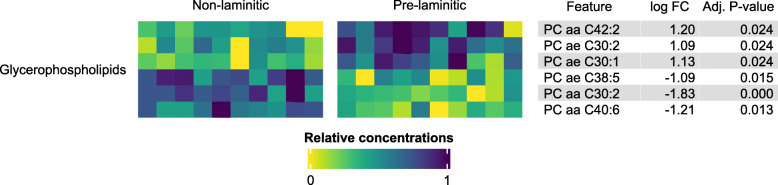
Heatmap illustrating the metabolites that differed significantly in concentration between pre-laminitic (PL) and non-laminitic (NL) ponies in postprandial samples. Three phosphatidylcholines (PC) were positively associated with eventual laminitis (darker colours in the “Pre-laminitic” column and positive log_2_ fold change [log FC]) while three were negatively associated with this condition. A log FC of 1 indicates that the normalized metabolite concentration was twice as high in PL in comparison to NL ponies

Postprandial samples collected after feeding the high-NSC diet resulted in a moderate separation in PCA between PL (*n* = 10) and NL (*n* = 10) ponies (Fig. 1b), and the differential concentration of six glycerophospholipids (Fig. 2, Additional file 2). Additionally, the glucose-alanine cycle was found to be enriched (false discovery rate [FDR] adjusted *P* = 0.048), with hexoses (H1), glutamic acid (Glu) and alanine (Ala) being positively associated with eventual laminitis.

#### Interaction between feeding and propensity for laminitis

In PCA (Fig. 1c) the basal and postprandial samples separated well along the first principal component, indicating that feeding alone explains a significant amount of variation within the data. A separation between PL (*n* = 5) and NL (*n* = 5) ponies was visible in the basal samples, but not in the postprandial samples. Thus, it appears that feeding induces opposite shifts along the second principal component for each group, and has the potential to influence the interpretation of metabolomic data in regard to laminitis risk.

**Fig. 3:**
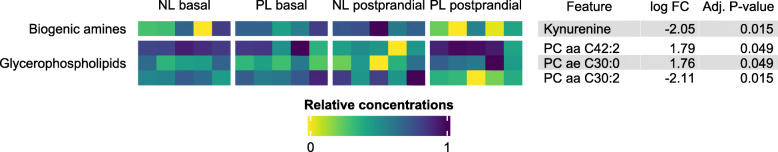
Heatmap illustrating one biogenic amine and three glycerophospholipids that showed a significant association with subsequent laminitis in samples collected from ponies before and after feeding. Kynurenine concentrations were low in basal samples and increased postprandially in non-laminitic (NL) ponies, but showed the opposite pattern in ponies that subsequently developed laminitis (PL). Log FC is the log_2_ fold change of the difference of differences between basal and postprandial samples from both PL and NL groups. The features are grouped by functional classes. Two features decrease in PL while increasing in NL postprandially (kynurenine and PC aa C30:2), while the other two (positive logFC) show an opposite pattern

As indicated by analysis of differential concentration, four metabolites were associated with group differences in the response to feeding. Kynurenine and PC aa C30:2 increased in the NL group after feeding, but decreased in PL ponies; whereas PC aa C42:2 and PC ae C30:0 displayed the opposite pattern (Fig. 3, Additional file 2). These differences were not associated with any significantly enriched metabolic pathway.

### Effect of PPID on the metabolic response to feeding

The separation of PPID (*n* = 6) and non-PPID (*n* = 14) ponies in PCA performed on the postprandial samples was weak to moderate, but also partly confounded by eventual laminitis (Additional file 3). No individual metabolites were affected significantly (Additional file 2) and no enriched pathways could be detected.

### Association between insulin and the metabolome in postprandial samples

**Fig. 4:**
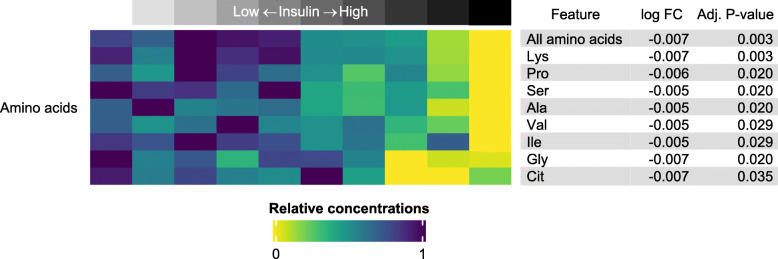
Heatmap illustrating the amino acids significantly associated with the insulin concentration in 10 non-laminitis ponies. All amino acids were present in lower concentrations in the ponies with the highest insulin concentrations. In the case of a linear predictor such as insulin, log FC indicates the variation in units of normalized amino acid concentration for every unit of insulin (in μIU/mL), hence the small values

The association between insulin concentrations and the metabolic profile was investigated in several ways. In a full model, including the factors of group (NL and PL), insulin and a group x insulin interaction, the metabolic differences between groups were not significant. Insulin, however, showed a significant negative association with 6 amino acids and with the sum of amino acids. None of the metabolites revealed any significant group x insulin interaction. In contrast, when a subgroup analysis was performed, two more amino acids (valine and isoleucine; see Fig. 4 and Additional file 2 for the complete list) were significant in NL ponies (*n* = 10), while no metabolites were significant in PL ponies (*n* = 10). Driven by the strong negative association between insulin and several amino acids in NL ponies, 15 significantly enriched pathways could be detected (Table 2).

**Table 2:** Metabolic pathways significantly enriched with increasing insulin concentrations. *P*-values are FDR adjusted. Metabolites included in each pathway are shown in bold when significantly contributing to the test result. The direction of association is indicated by (+) for a positive and (−) for a negative association with insulin. Despite being described as enrichment, variations in both directions are considered in absolute values, so that decreases in metabolite concentrations can result in significantly enriched pathways as well. Moreover, positive and negative associations within a pathway do not cancel each other out

Pathway	Adj. *P* value	Metabolites
Ammonia Recycling	0.001	**Ser** (−), **Gly** (−), **Asn** (−), **His** (−), Gln (−), Asp (−), Glu (+)
Glycine and Serine Metabolism	0.001	**Ser** (−), **Ala** (−), **Gly** (−), **Met** (−), Arg (−), Sarcosine (+), Glu (+)
Carnitine Synthesis	0.001	**Lys** (−), **Gly** (−), C0 (−)
Alanine Metabolism	0.002	**Ala** (−), **Gly** (−), Glu (+)
Urea Cycle	0.003	**Ala** (−), **Cit** (−), **Orn** (−), Arg (−), Gln (−), Asp (−), Glu (+)
Glutamate Metabolism	0.004	**Ala** (−), **Gly** (−), Gln (−), Asp (−), Glu (+)
Arginine and Proline Metabolism	0.004	**Pro** (−), **Gly** (−), **Cit** (−), **Orn** (−), Arg (−), Asp (−), Glu (+)
Valine, Leucine, and Isoleucine Degradation	0.009	**Val** (−), **Ile** (−), **Leu** (−), Glu (+)
Methionine Metabolism	0.011	**Ser** (−), **Gly** (−), **Met** (−), Met-SO (−), Putrescine (−), Sarcosine (+)
Glucose-Alanine Cycle	0.011	**Ala** (−), H1 (+), Glu (+)
Lysine Degradation	0.013	**Lys** (−), alpha-AAA (−), Glu (+)
Aspartate Metabolism	0.018	**Asn** (−), **Cit** (−), Arg (−), Gln (−), Asp (−), Glu (+)
Purine Metabolism	0.018	**Gly** (−), Gln (−), Asp (−), Glu (+)
beta-Alanine Metabolism	0.042	**His** (−), Carnosine (−), Asp (−), Glu (+)
Histidine Metabolism	0.045	**His** (−), Carnosine (−), Glu (+)

## Discussion

The present study investigated metabolic differences in blood samples from a previous experiment [10], which included ponies with varying degrees of ID. One group of ponies in this cohort developed laminitis upon being fed a high-NSC diet, and a second group did not. Both basal and postprandial samples taken before the ponies eventually developed laminitis were analysed, to determine the effect of feeding alone, and to explore any feeding x group interactions.

Although PCA analysis revealed an obvious difference between the groups both before and after feeding, the number of basal samples was small, and the laminitis groups could not be differentiated on the basis of individual metabolites, or pathways, using the basal samples alone. When the larger number of postprandial samples was analysed, individual metabolites and certain metabolic pathways were found to be associated with laminitis propensity. When investigating the feeding x group interaction, feeding alone had a marked effect on certain metabolites, masking the ability to differentiate between NL and PL ponies in postprandial samples using PCA alone. A considerable amount of variation in the data was explained by the variation in insulin concentrations. Unlike PL ponies, the postprandial metabolome of NL ponies displayed a negative association between amino acid and insulin concentrations. Lastly, no major impact on the metabolome due to the presence or absence of PPID could be detected.

### Metabolites and pathways potentially associated with a propensity for laminitis

While no single metabolites or pathways that differentiate NL from PL ponies could be identified in basal samples in the present study, previous equine metabolomic studies have identified differences using basal samples, associated with hyperinsulinemia [7, 8], obesity and laminitis history [8]. However, the number of significant metabolites reported varied based on the statistical test used, or was not adjusted for multiple comparisons, so it remains unclear if the absence of significant results in the present case is due to a lack of power, a different set of analysed metabolites, or the absence of substantial basal metabolic impact of a propensity for laminitis. Nevertheless, the fact that there was a notable separation between both groups in PCA, indicates they may be differentiated by a linear combination of metabolite concentrations (i.e. using multivariate statistical methods such as discriminant analysis).

In postprandial samples, three phosphatidylcholines (PCs) were increased in PL ponies, while three others were decreased. While the glycerophospholipid profile (including phosphatidylcholines) is known to be affected by insulin and hepatic metabolism, it remains difficult to associate single molecules with specific pathways or pathomechanisms [13]. Moreover, the lipids reported in the present study could be indicators of disease as well as of an accurate response to a different stimulus (e.g. higher insulin levels). Nevertheless, PC ae C38:5 was decreased in PL and has previously been reported to be negatively associated with body mass index in humans [14]. While neither cresty neck score (CNS) or body condition score (BCS) were significantly higher in PL ponies (data not shown), this result could be explained by a higher body fat content, which might have been underestimated by BCS [15]. In contrast to the down-regulation of PC aa C42:2 described in cows with hepatic lipidosis [16], this metabolite was increased in PL ponies, which, as explained above, may also be an indicator of an hepatic insulin sensitivity. Likewise, observed enrichment of the glucose-alanine cycle could result from an increase in glucose resorption, or the inhibition of gluconeogenesis [17]. Interestingly, an increased gluconeogenesis from alanine and lactate, potentially relying on similar mechanisms, was observed in type 2 diabetes mellitus in humans [18].

In humans, PCs with a higher degree of unsaturation (higher number of double bounds) appear to protect from diabetes progression, whereas an increased risk is associated with higher contents of saturated fatty acid chains [19, 20]. Similar conclusions were reached by Ding and Rexrode [21] regarding the risk for cardiovascular diseases. Interestingly, such polyunsaturated PCs (PC ae C38:5, PC aa C40:6; Fig. 2) were higher in NL than PL ponies in postprandial samples in the present study.

Phosphatidylcholine PC aa C42:2 was present in similar concentrations in PL and NL ponies in the basal state, but decreased upon feeding the high NSC diet in NL ponies, while increasing in the PL ponies. In contrast, kynurenine, previously reported to increase following an OGT in horses [7], displayed the opposite pattern. Because an enzyme activated during inflammation (indoleamine 2,3-dioxygenase, IDO) catalyses one pathway of kynurenine synthesis from tryptophan [22], an increase in kynurenine can be associated with low-grade inflammation as reported during the OGT in horses [7], in lame cows [23], and in humans with metabolic syndrome [5]. However, since neither tryptophan nor the kynurenine:tryptophan ratio showed a significant variation, the present results are not indicative of inflammation in either group. Overall, it should be noted that the basal (pre-feeding) and postprandial samples were collected a few days apart, and it is possible this may have affected the results. Additionally, the postprandial samples included in this study are not necessarily directly comparable to samples taken during an OGT. Further, an impact of the high NSC diet on the microbiome, itself affecting the metabolome, cannot be excluded, even if samples were taken at the beginning of the dietary challenge. Indeed, the influence of the microbiome on markers of the glucose/insulin homeostasis has previously been described in horses [24, 25].

The comparison between postprandial and basal samples in the first two principal components of PCA indicated that group differences were more obvious in the basal state than postprandially, suggesting that feeding a high NSC diet lessens the metabolic differences. However, one should bear in mind that the metabolites included in the panel were selected to reflect energy metabolism and are not exhaustive of the equine metabolome. In contrast, although this could be attributable to a larger sample size, both groups were distinguished by more metabolites in postprandial samples in univariate analysis. As a result, it cannot be concluded that either postprandial or basal testing is more powerful to predict eventual laminitis based on the present results. Yet, since dynamic tests are preferred for the diagnosis of ID because they exacerbate its impact on insulin secretion [26], the relationship between insulin and the postprandial metabolome is of high interest.

### Impact of insulin on the metabolome

A major and well described effect of insulin is the stimulation of amino acid uptake for protein synthesis, especially in muscle tissue [27]. During an OGT, amino acid concentrations in blood were shown to decrease in several species [7, 8, 28]; therefore the negative correlation between postprandial insulin and amino acid concentrations reported in the present study is expected. However, this effect was not detectable in PL ponies during subgroup analysis, suggesting some form of peripheral insulin resistance in this group. Since no group differences were found for amino acids when disregarding the insulin concentration, it is possible that this supposed insulin resistance was offset by hyperinsulinemia. To investigate this possibility further, the simultaneous assessment of ID and tissue-specific insulin sensitivity by immunoblotting [29], proteomics or hardly realisable (in horses) positron emission tomography in combination with a 18F fluorodeoxyglucose OGT [30] would be required.

The pathways associated with insulin are dominated by a restrained amino acid metabolism. Apart from glutamic acid (Glu), the results of this study further support enhanced protein synthesis. As blood insulin levels and BCS were slightly positively correlated (ρ = 0.37), it should be noted that Glu was positively associated with obesity in humans [31]. As a result, the (supposedly negative) effect of insulin on Glu may be offset by a positive effect of obesity.

## Conclusions

Previous metabolomic studies around EMS and ID in ponies and horses have described the response to OGT depending upon insulinemia [7, 8], obesity, and history of laminitis [8]. To the authors’ knowledge, however, this is the first study of the metabolome in relation to the future development of laminitis during a realistic dietary challenge. Six glycerophospholipids characterising the postprandial metabolome of PL ponies were identified as potential biomarkers for future risk of laminitis. Additionally, differences in the metabolic impact of insulin suggested that PL ponies have an insulin-sensitive hepatic metabolism, but insulin-resistant peripheral metabolism, which warrants further investigation on the proteomic level.

Due to the costs of such analyses, it was not possible to evaluate the predictive capabilities of these biomarkers (i.e. sensitivity and specificity) on additional samples in this study. Moreover, fewer samples were available for the differentiation of basal samples and to explore group x feeding interactions, negatively impacting the power of these analyses. Lastly, a genetic impact on the results cannot be excluded since only ponies were included in the present study. Further metabolomic investigations involving healthy, pre-laminitic and laminitic horses are warranted to clearly elucidate the prognostic value of these markers for laminitis in ponies with ID.

## Methods

The samples used in this study were obtained from a previous prospective trial approved by the Animal Care and Ethics Committees of Queensland University of Technology (Brisbane, Australia, #1400000575) and The University of Queensland (St Lucia, Australia, #QUT/SVS/114/14), which investigated laminitis occurrence in ponies with ID fed a high-NSC diet. The ponies were purchased from local owners and dealers. At the end of the trial, the ponies were offered for adoption and successfully rehomed. The samples were selected from a larger set based on the phenotype and insulin response of the ponies to an oral glucose test [10]. To know the details of the experiment that gave rise to this work consult [10].

### Animals

Due to cost constraints, subsets of samples were selected for metabolomic analysis to obtain balanced groups of eventually laminitic (*n* = 10) and non-laminitic (*n* = 10) ponies, based on their sex, dental age and diagnosis of PPID. At the time of sampling, none of the ponies had developed laminitis. Those who subsequently developed laminitis are classified here as PL; while those who remained sound are classed as NL. Basal samples were available in addition to the postprandial samples in five PL and five NL ponies, so that 30 samples were analysed in total. The characteristics of individual ponies in the sample population, including age, PPID status, season of sampling and insulin concentrations, are provided in an additional file (see Additional file 1).

### Basal samples

The basal samples were obtained by jugular venipuncture at 8 AM, after overnight fasting, between 1 and 5 days before the start of the dietary challenge period. Blood (10 mL) was collected into both plain serum tubes (for the analysis of insulin) and into EDTA-coated tubes (for the analysis of adrenocorticotropic hormone (ACTH) and metabolomic analysis). Blood in the serum tubes was allowed to clot for 20 min at room temperature, before centrifugation. Plasma tubes were centrifuged immediately. All samples were stored in 1 ml aliquots at − 80 °C.

### Postprandial samples

As described previously [10], during the dietary challenge period the ponies received a high-NSC diet of roasted-micronized oat flakes, lucerne (alfalfa) chaff, molasses and dextrose, which provided ∼12 g/NSC/kg bodyweight/d. The feed was divided into three daily meals and was given for up to 18 days. Lucerne hay and a vitamin/mineral supplement were provided additionally, as a separate meal, so that the total ration included ∼37% roughage. Postprandial samples were obtained on the morning of the second day of the challenge period, 90 min after feeding the first high-NSC meal. Blood was collected through a jugular catheter and processed as described above.

### Insulin measurements

Serum samples were transported on the day of collection to QML Pathology (Brisbane, Queensland, Australia) for the analysis of insulin concentrations using an ADVIA Centaur chemiluminescent assay (Siemens Healthcare Diagnostics, Bayswater, Victoria, Australia) [32].

### Diagnosis of PPID

The diagnosis or exclusion of PPID was based on a combination of clinical signs (hypertrichosis and polydipsia/polyuria) and basal ACTH concentrations using seasonally-adjusted cut-off values of > 27.8 pg/ml in non-autumn months and > 77.4 pg/ml in autumn months [33]. Adrenocorticotropic hormone levels were determined using the previously validated Immulite 2000 chemiluminescence method [34] by VetPath Laboratories (Ascot, Western Australia, Australia).

### Laminitis detection

All ponies were examined daily during the dietary challenge period. At the first indication of pain or lameness, a laminitis examination was conducted and filmed. The recording was sent immediately to two blinded experts for scoring on a 12 point scale, using a modification of the Obel method developed by Meier et al. [35]. A diagnosis of laminitis was made when the average score was > 3. In such cases, the pony was immediately removed from the diet and provided with the best standard of care for laminitis treatment, as previously described [10].

### Metabolomic assay

Metabolic profiling of the samples was performed at the Fraunhofer Institute of Toxicology and Experimental Medicine (ITEM, Hanover, Lower-Saxony, Germany) using a Biocrates AbsoluteIDQ p180 Kit (Biocrates Life Sciences AG, Innsbruck, Tyrol, Austria). This assay includes up to 188 metabolites related to glycolysis, oxidative processes, lipid degradation and inflammatory signalling. Amino acids and biogenic amines were measured by liquid chromatography (Agilent 1290 Infinity II LC, Santa Clara, CA, USA) tandem mass spectrometry (AB SCIEX 5500 QTrap mass spectrometer; AB SCIEX, Darmstadt, Germany), while acylcarnitines, hexoses, glycerophospholipids (PC and lysophosphatidylcholines), and sphingomyelins were quantified using flow injection analysis-tandem mass spectrometry.

### Statistical analysis

Statistical analysis was performed using R 4.0.2 [36]. The metabolomic dataset was prepared by removing metabolites when more than 50% of the values were below the limit of detection (LOD). Remaining values below the LOD were set to half the value of the LOD. After aligning QC samples by “QC robust LOESS signal correction” [37], metabolites with a coefficient of variation within QC-samples greater than 20% were removed as well. Missing values were imputed by the *k*-nearest neighbours method [38]. After log_2_-transformation, the data were scaled (auto-scale) and quantile normalised [39].

Analysis of differential concentration was performed with the ‘limma’ R-package [40]. In essence, unpaired *t*-tests with pooled variance moderated by empirical Bayes were performed for each metabolite, to investigate (1) metabolic differences between PL and NL ponies in basal and postprandial samples, as well as the feeding x group interaction (represented by the difference between postprandial and basal samples), and (2) the impact of PPID on the postprandial metabolome. The association between insulin and the postprandial metabolome (3) was analysed using a moderated linear model for each subgroup.

Metabolite set enrichment analysis was conducted for each of the three hypotheses using the ‘globaltest’ package [41]. In global tests, the alternative hypothesis is that a set of covariates (in this case, the metabolites of a pathway) is globally associated with an outcome, in a way that many weak associations can become significant. This approach relies on logistic regression for binary outcomes (eventual laminitis or PPID) and linear regression for continuous outcomes (insulin).

Principal component analysis was performed for each of the hypotheses. All reported *P*-values were adjusted to control a FDR of 5% using the Benjamini-Hochberg procedure [42].

## Supplementary Information

**Additional file 1: Table S1.** Characteristics of individual ponies in the sample population, including age, PPID status, season of sampling and insulin concentrations. Ten pre-laminitic (PL) and ten non-laminitic (NL) ponies were included in the study. Postprandial plasma samples were available from all ponies. Basal plasma samples were available for five NL and five PL ponies.

**Additional file 2: Table S2.** Top 10 metabolites from the linear models corresponding to each analysed hypothesis. The log_2_ fold change (logFC) is provided alongside its 95% confidence interval for each of the ten metabolites with lowest *p*-value. A positive logFC indicates higher metabolite concentrations in pre-laminitic or PPID ponies, or, for the feeding x group interaction, a stronger increase of metabolite concentrations in pre-laminitic ponies. *P*-values corresponding to a moderated *t*-statistic are given in addition to the FDR-adjusted *p-*value.

**Additional file 3: Fig. S1.** Principal component analysis from the post-prandial samples. Ponies with PPID are shown in yellow; ponies without PPID are shown in green. The 68% confidence ellipse for each group is represented in the corresponding colour.

## Data Availability

The dataset analysed during the current study is available from the corresponding author on reasonable request.

## Abbreviations

ACTH: Adrenocorticotropic hormone
Ala: Alanine
alpha-AAA: Alpha-Aminoadipic acid
Arg: Arginine
Asn: Asparagine
Asp: Aspartate
BCS: Body condition score
C0: Carnitine
Cit: Citrulline
EMS: Equine metabolic syndrome
FDR: False discovery rate
Gln: Glutamine
Glu: Glutamate
Gly: Glycine
H1: Sum of hexoses
His: Histidine
HMDB: Human metabolite database
ID: Insulin dysregulation
Ile: Isoleucine
Leu: Leucine
LOD: Limit of detection
Lys: Lysine
Met: Methionine
Met-SO: Methionine sulfoxide
MSEA: Metabolite set enrichment analysis
NL: Non-laminitic
NSC: Non-structural carbohydrate
OGT: Oral glucose test
Orn: Ornithine
PCA: Principal component analysis
PL: Pre-laminitic
PPID: Pituitary *pars intermedia* dysfunction
Pro: Proline
Ser: Serine
Val: Valine
