# Supplementary material for: Metabolic profile distinguishes laminitis-susceptible and -resistant ponies before and after feeding a high sugar diet
Source: BMC Vet Res. 2021 Jan 28;17:56. doi: 10.1186/s12917-021-02763-7 (PMC7841998; doi:10.1186/s12917-021-02763-7)
Supplement: Supplementary file 1 — Additional file 1: Table S1. Characteristics of individual ponies in the sample population, including age, PPID status, season of sampling and insulin concentrations. Ten pre-laminitic (PL) and ten non-laminitic (NL) ponies were included in the study. Postprandial plasma samples were available from all ponies. Basal plasma samples were available for five NL and five PL ponies. [file 12917_2021_2763_MOESM1_ESM.docx]

**Table S1. Characteristics of individual ponies in the sample population, including age, PPID status, season of sampling and insulin concentrations.** Ten pre-laminitic (PL) and ten non-laminitic (NL) ponies were included in the study. Postprandial plasma samples were available from all ponies. Basal plasma samples were available for five NL and five PL ponies.

| **Pony ID** | **Group** | **Sex** | **PPID** | **Dental age (yrs)** | **Height (cm)** | **Season of sampling** | **Basal insulin (µIU/ml)** | **Postprandial insulin (µIU/ml)** |
| --- | --- | --- | --- | --- | --- | --- | --- | --- |
| 034 | Pre-laminitic | Mare | no | 13 | 115 | Spring | 6 | 369 |
| 087 | Pre-laminitic | Gelding | yes | 16 | 93 | Autumn | 5 | 319 |
| 027 | Pre-laminitic | Gelding | yes | 15 | 114 | Autumn | 10 | 222 |
| 043 | Pre-laminitic | Gelding | yes | 22 | 102 | Summer | 8 | 205 |
| 023 | Pre-laminitic | Mare | no | 13 | 83 | Winter | 11 | 198 |
| 091 | Pre-laminitic | Mare | no | 10 | 82 | Summer |  | 583 |
| 002 | Pre-laminitic | Gelding | yes | 16 | 132 | Winter |  | 360 |
| 116 | Pre-laminitic | Gelding | yes | 20 | 122 | Autumn |  | 250 |
| 009 | Pre-laminitic | Mare | no | 20 | 123 | Winter |  | 180 |
| 003 | Pre-laminitic | Gelding | no | 10 | 102 | Winter |  | 160 |
| 030 | Non-laminitic | Mare | no | 10 | 122 | Spring | 7 | 169 |
| 042 | Non-laminitic | Gelding | no | 10 | 124 | Autumn | 6 | 76 |
| 133 | Non-laminitic | Gelding | no | 7 | 81 | Autumn | 4 | 62 |
| 033 | Non-laminitic | Gelding | no | 22 | 145 | Spring | 1 | 62 |
| 102 | Non-laminitic | Mare | yes | 20 | 81 | Autumn | 4 | 47 |
| 124 | Non-laminitic | Gelding | no | 18 | 102 | Autumn |  | 440 |
| 071 | Non-laminitic | Mare | no | 15 | 142 | Autumn |  | 366 |
| 029 | Non-laminitic | Gelding | no | 16 | 145 | Spring |  | 268 |
| 064 | Non-laminitic | Mare | no | 11 | 125 | Autumn |  | 195 |
| 127 | Non-laminitic | Mare | no | 10 | 91 | Autumn |  | 81 |
