# Supplementary material for: Metabolic profile distinguishes laminitis-susceptible and -resistant ponies before and after feeding a high sugar diet
Source: BMC Vet Res. 2021 Jan 28;17:56. doi: 10.1186/s12917-021-02763-7 (PMC7841998; doi:10.1186/s12917-021-02763-7)
Supplement: Supplementary file 2 — Additional file 2: Table S2. Top 10 metabolites from the linear models corresponding to each analysed hypothesis. The log2 fold change (logFC) is provided alongside its 95% confidence interval for each of the ten metabolites with lowest p-value. A positive logFC indicates higher metabolite concentrations in pre-laminitic or PPID ponies, or, for the feeding x group interaction, a stronger increase of metabolite concentrations in pre-laminitic ponies. P-values corresponding to a moderated t-statistic are given in addition to the FDR-adjusted p-value. [file 12917_2021_2763_MOESM2_ESM.docx]

**Table S2. Top 10 metabolites from the linear models corresponding to each analysed hypothesis.** The log_2_ fold change (logFC) is provided alongside its 95% confidence interval for each of the ten metabolites with lowest p-value. A positive logFC indicates higher metabolite concentrations in pre-laminitic or PPID ponies, or, for the feeding x group interaction, a stronger increase of metabolite concentrations in pre-laminitic ponies. P-values corresponding to a moderated t-statistic are given in addition to the FDR-adjusted p-value.

| **Metabolite** | **Class** | **HMDB ID** | **logFC** | **CI.low** | **CI.upp** | **t** | **P value** | **adj.P val** |
| --- | --- | --- | --- | --- | --- | --- | --- | --- |
| **Basal top 10** |  |  |  |  |  |  |  |  |
| lysoPC a C18:1 | glycerophospholipids | HMDB0002815 | 1,482 | 0,587 | 2,377 | 3,399 | 0,002 | 0,149 |
| lysoPC a C16:0 | glycerophospholipids | HMDB0010382 | 1,547 | 0,607 | 2,487 | 3,378 | 0,002 | 0,149 |
| lysoPC a C18:0 | glycerophospholipids | HMDB0010384 | 1,461 | 0,519 | 2,402 | 3,184 | 0,004 | 0,150 |
| PC aa C30:0 | glycerophospholipids | HMDB0011203 | -1,270 | -2,113 | -0,426 | -3,090 | 0,005 | 0,150 |
| lysoPC a C18:2 | glycerophospholipids | HMDB0010386 | 1,171 | 0,323 | 2,020 | 2,834 | 0,009 | 0,150 |
| Leu | amino acids | HMDB0000687 | 1,274 | 0,351 | 2,198 | 2,833 | 0,009 | 0,150 |
| lysoPC a C16:1 | glycerophospholipids | HMDB0010383 | 1,180 | 0,315 | 2,044 | 2,802 | 0,009 | 0,150 |
| SM C18:0 | sphingolipids | HMDB0012087 | -1,263 | -2,196 | -0,330 | -2,779 | 0,010 | 0,150 |
| biogenic amines | biogenic amines |  | -1,173 | -2,044 | -0,302 | -2,763 | 0,010 | 0,150 |
| lysoPC a C17:0 | glycerophospholipids | HMDB0012108 | 1,141 | 0,228 | 2,054 | 2,565 | 0,016 | 0,201 |
| **Post-prandial top 10** | |  |  |  |  |  |  |  |
| PC aa C30:2 | glycerophospholipids | HMDB0007999 | -1,829 | -2,459 | -1,200 | -5,943 | 0,000 | 0,000 |
| PC aa C40:6 | glycerophospholipids | HMDB0008121 | -1,206 | -1,787 | -0,625 | -4,244 | 0,000 | 0,013 |
| PC ae C38:5 | glycerophospholipids | HMDB0013432 | -1,086 | -1,632 | -0,540 | -4,063 | 0,000 | 0,015 |
| PC ae C30:2 | glycerophospholipids | HMDB0013410 | 1,091 | 0,492 | 1,690 | 3,725 | 0,001 | 0,024 |
| PC aa C42:2 | glycerophospholipids | HMDB0008092 | 1,200 | 0,525 | 1,875 | 3,635 | 0,001 | 0,024 |
| PC ae C30:1 | glycerophospholipids | HMDB0013402 | 1,134 | 0,494 | 1,774 | 3,623 | 0,001 | 0,024 |
| PC ae C30:0 | glycerophospholipids | HMDB0013341 | 1,122 | 0,361 | 1,882 | 3,013 | 0,005 | 0,088 |
| PC aa C38:6 | glycerophospholipids | HMDB0008147 | -0,957 | -1,607 | -0,307 | -3,009 | 0,005 | 0,088 |
| SM C22:3 | sphingolipids | HMDB0013468 | -0,820 | -1,399 | -0,241 | -2,894 | 0,007 | 0,104 |
| PC aa C38:1 | glycerophospholipids | HMDB0008269 | -0,864 | -1,495 | -0,233 | -2,798 | 0,009 | 0,119 |
| **Feeding x Group interaction top 10** | |  |  |  |  |  |  |  |
| PC aa C30:2 | glycerophospholipids | HMDB0007999 | -2,114 | -3,151 | -1,078 | -4,077 | 0,000 | 0,015 |
| Kynurenine | biogenic amines | HMDB0000684 | -2,050 | -3,097 | -1,003 | -3,914 | 0,000 | 0,015 |
| PC ae C30:0 | glycerophospholipids | HMDB0013341 | 1,757 | 0,701 | 2,812 | 3,327 | 0,001 | 0,049 |
| PC aa C42:2 | glycerophospholipids | HMDB0008092 | 1,793 | 0,715 | 2,872 | 3,324 | 0,001 | 0,049 |
| Kyn/Trp |  |  | -1,658 | -2,729 | -0,587 | -3,095 | 0,003 | 0,078 |
| Gly | amino acids | HMDB0000123 | -1,611 | -2,706 | -0,516 | -2,941 | 0,005 | 0,101 |
| Thr | amino acids | HMDB0000167 | -1,445 | -2,524 | -0,366 | -2,678 | 0,009 | 0,171 |
| lysoPC a C18:1 | glycerophospholipids | HMDB0002815 | -1,361 | -2,411 | -0,312 | -2,593 | 0,012 | 0,171 |
| amino acids | amino acids |  | -1,367 | -2,428 | -0,305 | -2,574 | 0,012 | 0,171 |
| PC aa C30:0 | glycerophospholipids | HMDB0011203 | 1,323 | 0,276 | 2,370 | 2,526 | 0,014 | 0,171 |
| **Insulin top 10** |  |  |  |  |  |  |  |  |
| All amino acids | amino acids |  | -0,007 | -0,009 | -0,004 | -6,018 | 0,000 | 0,003 |
| Lys | amino acids | HMDB0000182 | -0,007 | -0,010 | -0,004 | -5,693 | 0,000 | 0,003 |
| Ser | amino acids | HMDB0000187 | -0,005 | -0,008 | -0,003 | -4,308 | 0,001 | 0,020 |
| Ala | amino acids | HMDB0000161 | -0,005 | -0,008 | -0,003 | -4,219 | 0,001 | 0,020 |
| Gly | amino acids | HMDB0000123 | -0,007 | -0,010 | -0,003 | -4,190 | 0,001 | 0,020 |
| Pro | amino acids | HMDB0000162 | -0,006 | -0,008 | -0,003 | -4,094 | 0,001 | 0,020 |
| Ile | amino acids | HMDB0000172 | -0,005 | -0,009 | -0,002 | -3,778 | 0,002 | 0,029 |
| Val | amino acids | HMDB0000883 | -0,005 | -0,007 | -0,002 | -3,772 | 0,002 | 0,029 |
| Cit | amino acids | HMDB0000904 | -0,007 | -0,011 | -0,003 | -3,623 | 0,002 | 0,035 |
| PC ae C34:3 | glycerophospholipids | HMDB0013413 | 0,003 | 0,001 | 0,006 | 3,190 | 0,006 | 0,078 |
| **PPID top 10** |  |  |  |  |  |  |  |  |
| PC aa C30:2 | glycerophospholipids | HMDB0007999 | -1,124 | -2,059 | -0,188 | -2,457 | 0,020 | 0,668 |
| PC aa C34:4 | glycerophospholipids | HMDB0007884 | -0,695 | -1,299 | -0,091 | -2,353 | 0,026 | 0,668 |
| sphingolipids | sphingolipids |  | -0,795 | -1,505 | -0,085 | -2,289 | 0,030 | 0,668 |
| Orn | amino acids | HMDB0000214 | 1,053 | 0,108 | 1,997 | 2,279 | 0,030 | 0,668 |
| Sarcosine | biogenic amines | HMDB0000271 | 1,079 | 0,090 | 2,067 | 2,232 | 0,033 | 0,668 |
| PC ae C36:2 | glycerophospholipids | HMDB0013418 | -0,642 | -1,239 | -0,046 | -2,202 | 0,036 | 0,668 |
| PC ae C32:1 | glycerophospholipids | HMDB0013404 | -0,749 | -1,466 | -0,032 | -2,137 | 0,041 | 0,668 |
| lysoPC a C18:0 | glycerophospholipids | HMDB0010384 | 0,605 | 0,013 | 1,197 | 2,090 | 0,046 | 0,668 |
| H1 | sugars | HMDB0000122 | 0,686 | -0,001 | 1,373 | 2,043 | 0,050 | 0,668 |
| Gln | amino acids | HMDB0000641 | 0,697 | -0,002 | 1,396 | 2,039 | 0,051 | 0,668 |
