# Supplementary figures and images for: Metabolic profile distinguishes laminitis-susceptible and -resistant ponies before and after feeding a high sugar diet
Source: BMC Vet Res. 2021 Jan 28;17:56. doi: 10.1186/s12917-021-02763-7 (PMC7841998; doi:10.1186/s12917-021-02763-7)

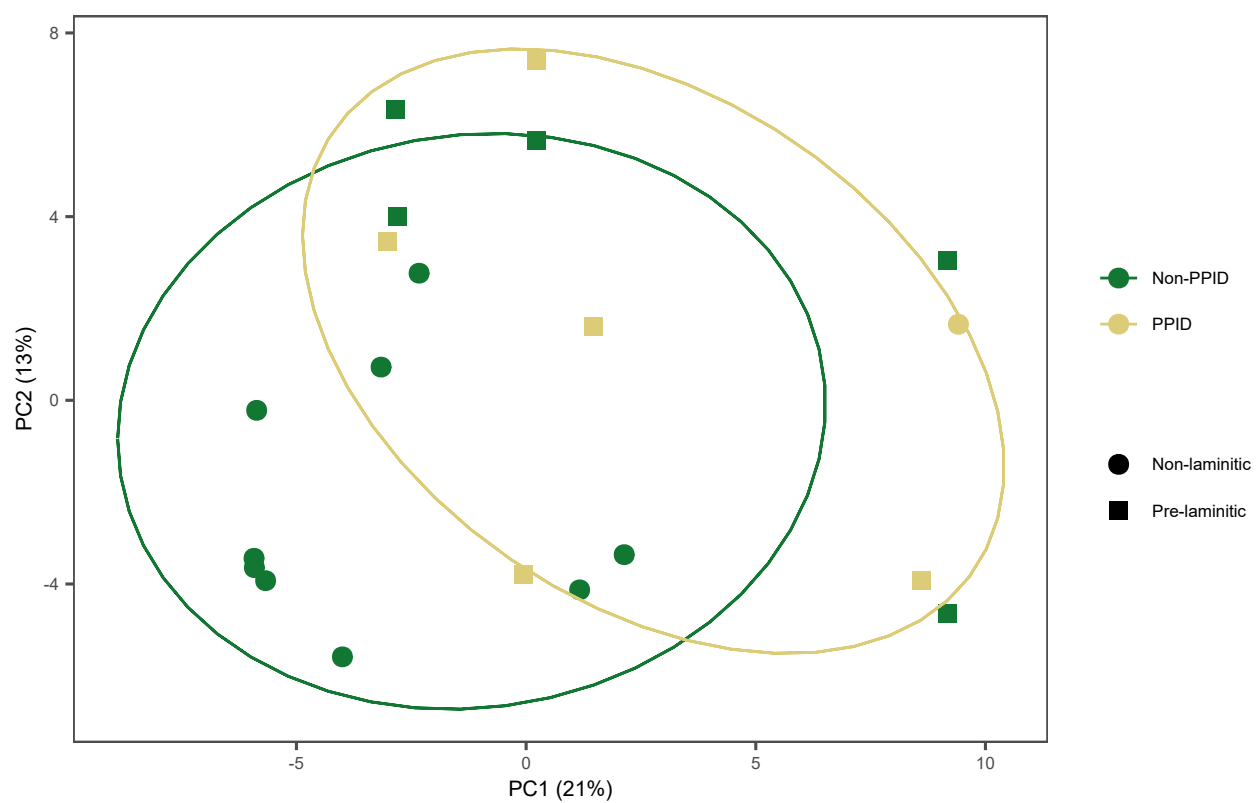

Supplement: Supplementary file 3 — Additional file 3: Fig. S1. Principal component analysis from the post-prandial samples. Ponies with PPID are shown in yellow; ponies without PPID are shown in green. The 68% confidence ellipse for each group is represented in the corresponding colour. [file 12917_2021_2763_MOESM3_ESM.pdf]
